# Impact of Data Distribution and Bootstrap Setting on Anomaly Detection Using Isolation Forest in Process Quality Control

**DOI:** 10.3390/e27070761

**Published:** 2025-07-18

**Authors:** Hyunyul Choi, Kihyo Jung

**Affiliations:** 1 School of Industrial and Management Engineering, Korea University, Seoul 02841, Republic of Korea; hyunyul@korea.ac.kr; 2 School of Industrial Engineering, University of Ulsan, Ulsan 44610, Republic of Korea

**Keywords:** isolation forest, novelty detection, non-normal distributions, multivariate data, statistical process control, unsupervised learning

## Abstract

This study investigates the impact of data distribution and bootstrap resampling on the anomaly detection performance of the Isolation Forest (iForest) algorithm in statistical process control. Although iForest has received attention for its multivariate and ensemble-based nature, its performance under non-normal data distributions and varying bootstrap settings remains underexplored. To address this gap, a comprehensive simulation was performed across 18 scenarios involving log-normal, gamma, and *t*-distributions with different mean shift levels and bootstrap configurations. The results show that iForest substantially outperforms the conventional Hotelling’s T^2^ control chart, especially in non-Gaussian settings and under small-to-medium process shifts. Enabling bootstrap resampling led to marginal improvements across classification metrics, including accuracy, precision, recall, F1-score, and average run length (ARL)_1_. However, a key limitation of iForest was its reduced sensitivity to subtle process changes, such as a 1σ mean shift, highlighting an area for future enhancement.

## 1. Introduction

Anomaly detection has been widely studied and applied in quality engineering, where process variability and stability are monitored using statistical process control (SPC) charts. Modern SPC combines traditional control charts based on statistical theory with novelty detection algorithms. A key objective in modern SPC is to identify abnormalities in out-of-control processes as early as possible to prevent the production of defective products. Traditional SPC charts are either univariate, monitoring quality characteristics independently, or multivariate, which monitor interrelated quality characteristics simultaneously.

The Shewhart $\bar{Χ}$-chart [1] is an effective univariate control chart for detecting large shifts in process parameters. However, because it only considers current sample data, it does not utilize past information. It is also inefficient in detecting small process shifts, such as those within 1.5 sigma. To address this, the cumulative sum (CUSUM) [2] and exponentially weighted moving average (EWMA) [3] control charts were introduced. EWMA effectively detects small changes and long-term trends by assigning more weight (λ) to recent data, with earlier values weighted by a decreasing exponential sequence: λ(1−λ), $\lambda {\left(1-\lambda \right)}^{2}$, … (0≤λ≤1, and so on (0 ≤ λ ≤ 1). When λ = 1, EWMA is equivalent to the $\bar{Χ}$-chart. Similarly, CUSUM is effective for detecting minor process changes by accumulating quality metrics over time. It quickly identifies shifts by synthesizing continuous sample data, making it well-suited for identifying small deviations in controlled processes.

Unlike univariate control charts, multivariate charts can monitor multiple quality characteristics simultaneously, such as Hotelling’s T^2^ [4], multivariate CUSUM [5], and multivariate EWMA [6] control charts. The robustness of the multivariate EWMA chart to non-normality has been examined [7]. Shewhart-type distribution-free control charts based on Wilcoxon signed-rank statistics and run-type rules were proposed in [8]. A multivariate non-parametric control chart based on a bivariate sign test was introduced in [9]. Recent studies have also developed non-parametric principal component analysis (PCA) control charts that do not require distributional assumptions [10]. A non-parametric control chart capable of adaptively monitoring time-varying and multimodal processes was presented in [11].

Existing novelty detection methods can be broadly categorized into three types: density-based, distance/reconstruction-based, and model-based. The expectation-maximization (EM) algorithm [12] is a density-based method consisting of an E-step, which computes conditional probabilities based on current parameter estimates, and an M-step, which updates parameters to maximize expected likelihood. In [13], the in-control performance of the phase I T^2^ chart was assessed by estimating missing values using four imputation techniques, including the EM algorithm. The local outlier factor (LOF) algorithm [14] is another density-based method that computes novelty scores based on local data density. The k-nearest neighbors (KNN) algorithm [15,16] determines novelty scores by measuring distances to the k-nearest neighbors without assuming prior distributions for the normal class. Among distance-based approaches, the k-means clustering method [17] calculates novelty scores using distance to the nearest centroid, also without requiring prior distributional assumptions. Autoencoder-based methods [18] can also be applied to novelty detection by nonlinear transformation and latent space representations. One-class support vector machine (1-SVM) [19] and support vector data description (SVDD) [20] are widely used model-based novelty detection techniques. The 1-SVM method maps data into a feature space defined by the kernel and separates them from the origin with a maximum margin. SVDD identifies a hypersphere that encloses all normal instances within the feature space. The Isolation Forest (iForest) algorithm, developed in [21], is another model-based novelty detection technique. It differs fundamentally by explicitly isolating anomalies rather than profiling normal points. Additionally, graph-based methods such as spectral clustering with graph structure learning [22] can be applied to novelty detection by leveraging network science concepts to capture topological dependencies among features.

Among the novelty detection methods mentioned above, the iForest algorithm has undergone several improvements and adaptations since its introduction in [21]. The SA-iForest and Extended-iForest variants were proposed in [23,24], and iForest was compared with neighbor-based isolation methods in [25]. These developments have progressively enhanced iForest’s performance. However, a significant gap remains in the analysis of non-normal data distributions—an essential aspect of data analytics [26] and a common characteristic of industrial processes. Moreover, the influence of the bootstrap setting—determining whether data sampling during tree construction occurs with or without replacement, has not been extensively studied. This setting can affect the diversity of isolation trees and, in turn, the model’s robustness and generalization performance. Despite its potential importance, the impact of bootstrap configuration on iForest remains largely unexplored.

This study systematically examines the effects of data distribution and bootstrap setting on the performance of iForest in process quality control. The remainder of this paper is structured as follows. Section 2 reviews the two algorithms (iForest and T^2^) evaluated in this study. Section 3 details the methodology used to assess their performance. Section 4 discusses the performance of iForest under varying data distributions and bootstrap settings in comparison with T^2^. Finally, Section 5 summarizes key findings, discusses practical implications, and outlines potential applications in process quality control.

## 2. Algorithm Review

### 2.1. iForest Algorithm

**Figure 1:**
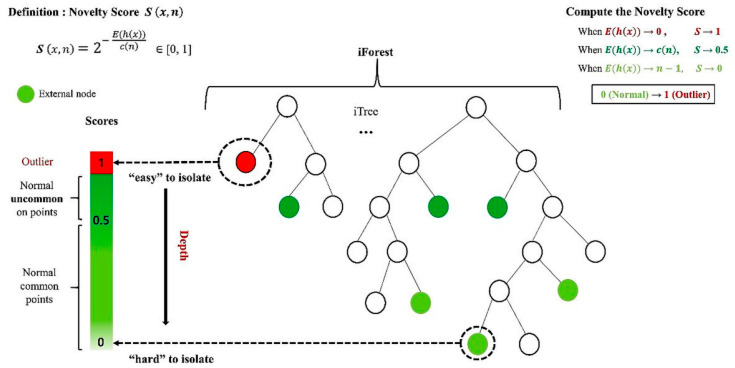
Schematic of isolation forest.

In the iForest algorithm, random decision trees (called iTrees) are constructed until each instance in the dataset is isolated in a separate leaf. This process is repeated multiple times, and the average path length is used to compute a novelty score ranging from 0 to 1 (0 for normal instances and 1 for anomalies), as illustrated in Figure 1.

The core objective of iForest is to isolate individual observations by randomly selecting features and corresponding split values within their respective data ranges. “Isolation” refers to the separation of a given instance from the rest of the dataset, resulting in the creation of iTrees. Each node in an iTree represents a collection of vectors forming a subset of the original dataset, which consists of N-dimensional points. In iTrees, instances are recursively split until each one is isolated.

iTrees are built through a three-step random partitioning process. First, a subsample of size *n*, denoted as *X*′, is generated using bootstrapping from the original dataset *X*, which contains *N* instances with *k*-dimensional features. Second, *X*′ is recursively divided by randomly selecting a feature *q* and a split value *p* to construct the iTree. This continues until specific stopping conditions are met, such as reaching a maximum tree height or having only one instance in a node. Third, the procedure is repeated *m* times to generate *m* iTrees. Assuming all instances are distinct, each instance is isolated at an external node when the iTree is fully developed. In this case, the number of external nodes is *n*, and the number of internal nodes is *n* − 1. Therefore, each iTree contains a total of 2*n* − 1 nodes [27].

Instances with short average path lengths are likely to be outliers. The path length *h*(*x*) of a given instance *x* is defined by the number of edges traversed in an iTree from the root to the external node where the search ends. However, the average path length is influenced by the sample size *n*. To account for this, *h*(*x*) is normalized using the theoretical average path length. The estimated average path length c(n) is computed using Equation (1):

(1) $$c\left(n\right)=2H\left(n-1\right)-(\frac{2\left(n-1\right)}{n}),$$

where $H\left(i\right)$ denotes a harmonic number, which can be estimated by $H\left(i\right)=\ln\left(i\right)+0.5772156649,$ and n denotes the number of instances.

The novelty score, denoted as the normalized score S$\left(x,n\right)$, is calculated using Equation (2). Instances with *S* values close to 1 are clearly anomalies, while those with *S* values near 0, particularly below 0.5, are classified as normal [27].

(2) $$S\left(x,n\right)=2^{-\frac{E(h\left(x\right))}{c\left(n\right)}}\;\in \;[0,1]$$

where *E*(*h*(*x*)) is the average path length of instance *x*, and *c*(*n*) is the estimated average path length.

### 2.2. Hotelling’s T^2^

Hotelling’s T^2^ statistic is a multivariate extension of the Student’s *t*-test used to assess whether the mean vector of a multivariate sample significantly differs from a specified or known mean. It is widely employed in multivariate process monitoring for quality control applications, particularly when relationships among multiple correlated variables must be considered simultaneously. The T^2^ statistic incorporates the covariance structure among variables, making it more robust than univariate control charts for detecting multivariate shifts. Its formulation ensures that outlier detection reflects the overall Mahalanobis distance in multivariate space rather than being skewed by marginal distributions.

Given a multivariate sample $x_{1},x_{2},\ldots ,x_{n}\in R^{p},$ where $\bar{x}$ is the sample mean vector and ∑ is the population covariance matrix, the T^2^ statistic for testing the mean vector $\mu_{0}$ is defined as

(3) $$T^{2}=n{\left(\bar{x}-\mu_{0}\right)}^{T}S^{-1}(\bar{x}-\mu_{0})$$

where $\bar{x}=\frac{1}{n}\sum_{i=1}^{n}x_{i}$ is the sample mean vector, $S=\frac{1}{n-1}\sum_{i=1}^{n}\left(x_{i}-\bar{x}\right){\left(x_{i}-\bar{x}\right)}^{T}$ is the sample covariance matrix, *n* is the sample size, and *p* is the number of variables.

Under the null hypothesis *H*_0_:$\bar{x}$ = $\mu_{0}$ and assuming multivariate normality, the T^2^ statistic follows an exact F-distribution:

(4) $$\frac{n-p}{p(n-1)}T^{2}\sim F_{p,n-p},$$

The T^2^ statistic offers several advantages in multivariate analysis. Unlike univariate tests, it accounts for the correlation structure among variables, enhancing sensitivity to joint anomalies. It is based on Mahalanobis distance, which measures the deviation from the multivariate while incorporating both variance and covariance. Its validity depends on the assumption of multivariate normality, under which reliable inference can be drawn. Due to these properties, T^2^ is widely used in multivariate SPC (MSPC), particularly in Phase I for establishing baseline control limits and in Phase II for identifying deviations that indicate abnormal process behavior.

## 3. Material and Methods

### 3.1. Evaluation Scenarios

Eighteen scenarios were designed based on combinations of non-normal distributions (log-normal, gamma, and *t*-distribution) and a bootstrap-related hyperparameter. These three distribution types, frequently used in prior studies [28,29,30], were selected to reflect diverse data patterns commonly observed in manufacturing processes. The bootstrap setting included two levels: false and true. When set to true, individual iTrees were trained on random subsets of the data sampled with replacement. When set to false, sampling was performed without replacement.

**Figure 2:**
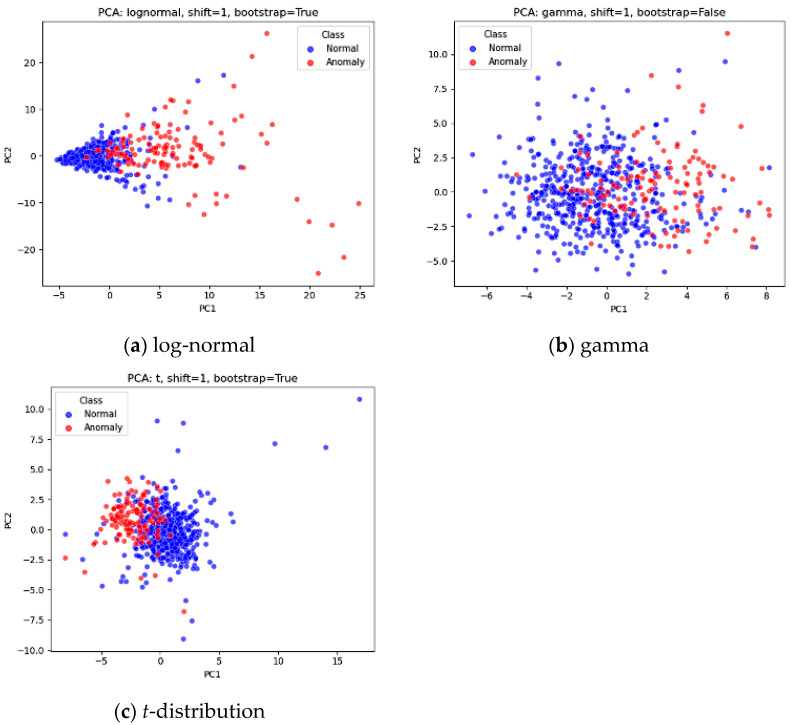
Examples of normal and anomaly (1 mean shift) data for three distributions (note: two principal components extracted for the 10 attributes were used for visualization purpose).

Datasets were generated using log-normal, gamma, and *t*-distributions, as illustrated in Figure 2. For Phase I analysis, 500 in-control observations with 10 attributes were generated following prior research [21,31,32]. For Phase II analysis, 100 out-of-control observations were created to simulate three levels of mean shift in manufacturing processes (1, 2, and 3 corresponding to small, medium, and large shifts, respectively). For the *t*-distribution, the degrees of freedom were set to three for both Phase I (in-control) and Phase II (out-of-control), which is in line with previous studies [33,34]. The log-normal distribution represented skewed process data, the gamma distribution captured skewness and high kurtosis, and the *t*-distribution reflected symmetry with high kurtosis.

### 3.2. Control Limits

Control limits were set based on the average run length (ARL), defined as the average number of observations required to detect a process change [35]. To determine the control limits for iForest, anomaly scores were computed using an ensemble of 100 iTrees for each scenario. To ensure reliable and stable results—given the stochastic nature of iForest subsampling—this process was repeated 10 times. Since control charts are expected to provide early warnings when a process change occurs, a smaller out-of-control ARL (${ARL}_{1}$), given a fixed in-control ARL (${ARL}_{0}$ = 370, equivalent to 3 sigma level), allows for more accurate and timely detection of abnormal conditions [36]. ${ARL}_{0}$ refers to the expected number of samples until a signal is triggered while the process remains in control. Conversely, ${ARL}_{1}$ is the expected number of samples until a signal is triggered when the process is actually out of control. Finally, the control limits for T^2^ were calculated using the same procedure as that used for iForest.

### 3.3. Performance Measures

Appropriate use of evaluation metrics is critical for assessing model performance and guiding model selection in binary classification tasks. We used six evaluation metrics: accuracy, precision, recall (true positive rate, TPR), F1-score (F1), ROC area under the curve (AUC) (receiver operating characteristic—area under the curve), and PR AUC (precision–recall area under the curve). Accuracy measures overall classification correctness, representing the proportion of correctly predicted instances (both positive and negative) among all observations. It is calculated as TP + TN/(TP + TN + FP + FN). Although intuitive, accuracy can be misleading for imbalanced datasets, as they may be dominated by the majority class. Precision is the proportion of true positives among all positive predictions made by the model. It reflects the model’s ability to minimize false positives and is calculated as TP/(TP + FP). Precision is especially important when false positives carry a high cost. Recall indicates the model’s ability to correctly identify all actual positives. It is calculated as: TP/(TP + FN). Recall is crucial in applications where missing positive cases (false negatives) has serious consequences, such as in medical diagnostics or fault detection. False positive rate (FPR) represents the proportion of actual negatives incorrectly classified as positives. It is calculated as FP/(FP + TN). F1-score balances precision and recall and is particularly useful when an even trade-off is needed. It is calculated as

(5) $$F1=\frac{2\times precision\times recall}{presion+recall}$$

The ROC AUC quantifies a classifier’s overall ability to distinguish between positive and negative classes, regardless of the decision threshold. The ROC curve plots the TPR against the FPR. The AUC represents the probability that a randomly selected positive instance is ranked higher than a randomly selected negative one by the classifier. AUC values range from 0.5 (no discrimination) to 1.0 (perfect discrimination). The PR AUC represents the area under the precision–recall curve, which plots precision against recall across various threshold settings. It provides a comprehensive summary of model performance, which is particularly useful in imbalanced datasets. A higher PR AUC indicates a better trade-off between precision and recall. Unlike ROC AUC, PR AUC focuses solely on performance with respect to the positive class, making it more informative in skewed settings.

### 3.4. Statistical Analysis

A three-factor analysis of variance (ANOVA) was conducted to examine the effects of distribution type, mean shift level, and bootstrap setting, using a significance level of 0.05. For factors and interactions found to be significant, post hoc analyses were performed using Tukey’s test and simple effect tests at the same significance level.

## 4. Results

### 4.1. Effect of Distributions, Mean Shifts, and Bootstrap Settings in iForest

**Figure 3:**
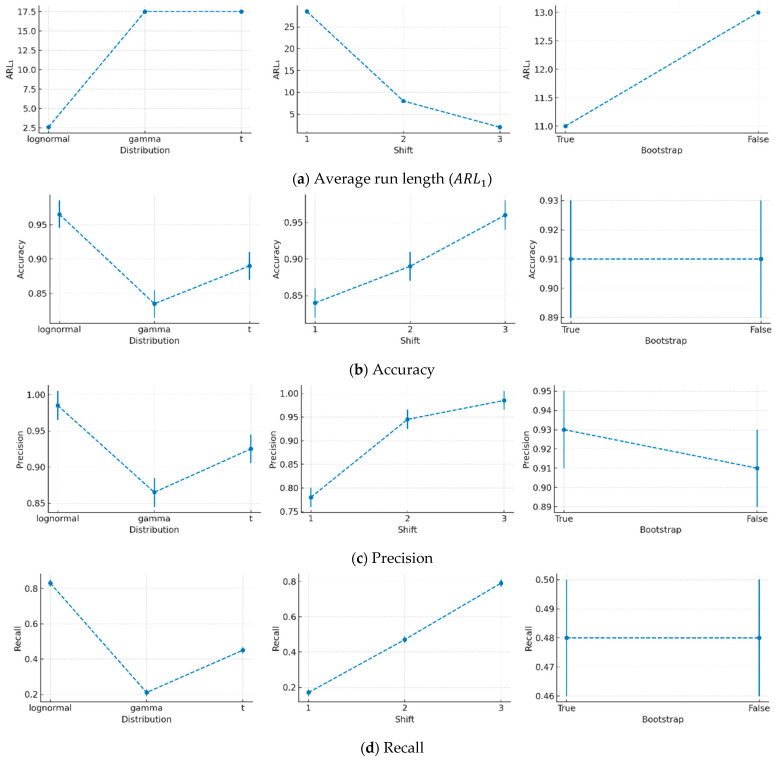

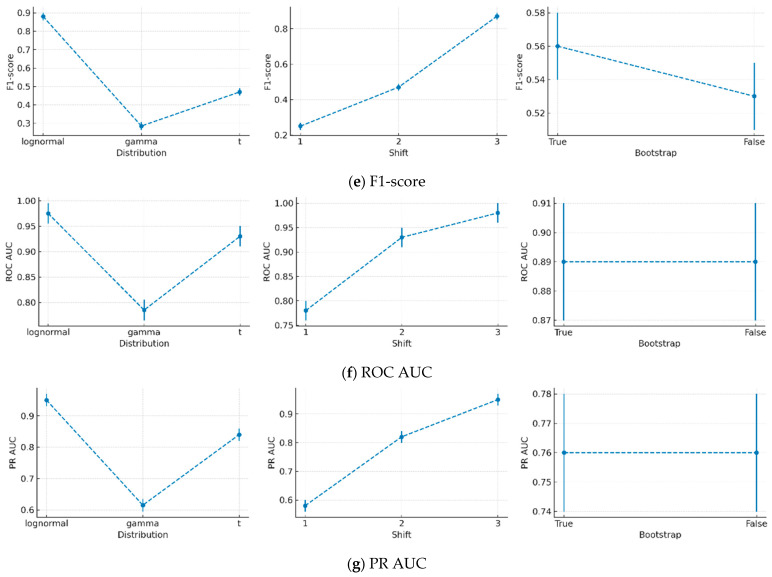
Effect of data distribution, mean shift, and bootstrap setting in iForest.

Two out of three factors—distribution type (*F*(2, 156) = 37.2, *p* < 0.001), mean shift (*F*(2, 156) = 73.3, *p* < 0.001), and bootstrap setting (*F*(1, 156) = 0.1, *p* = 0.74)—had a significant effect on ${ARL}_{1}$, as shown in Figure 3a. Among the distributions, the log-normal distribution yielded the lowest *ARL*_1_ (mean ± SD; 1.6 ± 0.9), indicating superior detection performance. In contrast, the gamma (18.2 ± 24.4) and *t*-distributions (16.4 ± 27.3) produced similar ${ARL}_{1}$ values. The mean shift, which reflects the degree of separation between in-control and out-of-control data, significantly reduced ${ARL}_{1}$ as its level increased. However, the reduction in ${ARL}_{1}$ diminished with each successive shift level—from 23.6 to 7.4 between shift levels 1 and 2 and from 7.4 to 1.7 between levels 2 and 3. Although the bootstrap setting had an insignificant effect, enabling bootstrap resulted in a slight difference between the True (8.3 ± 13.3) and False (11.7 ± 23.4) conditions.

All classification performance metrics followed consistent trends across the three factors, as shown in Figure 3b–g. The log-normal distribution produced the highest values for all metrics compared to the other distributions. While the gamma and *t*-distributions showed similar performance, the *t*-distribution slightly outperformed the gamma distribution. Increasing the mean shift significantly improved all metrics, and this trend remained consistent across all evaluation measures. Lastly, the true bootstrap setting yielded slightly better performance than false across all metrics.

**Figure 4:**
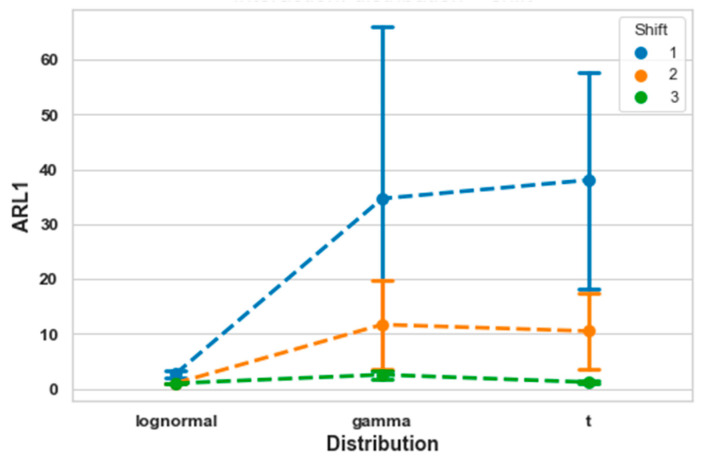
Interaction between distribution and mean shift for ${ARL}_{1}$.

Significant interactions were observed between distribution type and mean shift for all metrics (accuracy: *F*(4, 156) = 134.9, *p* < 0.001; precision: *F*(4, 156) = 69.7, *p* < 0.001; recall: *F*(4, 156) = 134.9, *p* < 0.001; F1-score: *F*(4, 156) = 82.4, *p* < 0.001; ROC AUC: *F*(4, 156) = 509.1, *p* < 0.001; PR AUC: *F*(4, 156) = 422.6, *p* < 0.001). For instance, as shown in Figure 4 for ${ARL}_{1}$, the log-normal distribution remained stable across different mean shifts. In contrast, for the gamma and *t*-distributions, ${ARL}_{1}$ increased substantially as the mean shift decreased. This indicates that small process shifts are more difficult to detect when the data follow these distributions.

### 4.2. Comparison of iForest with T^2^

**Figure 5:**
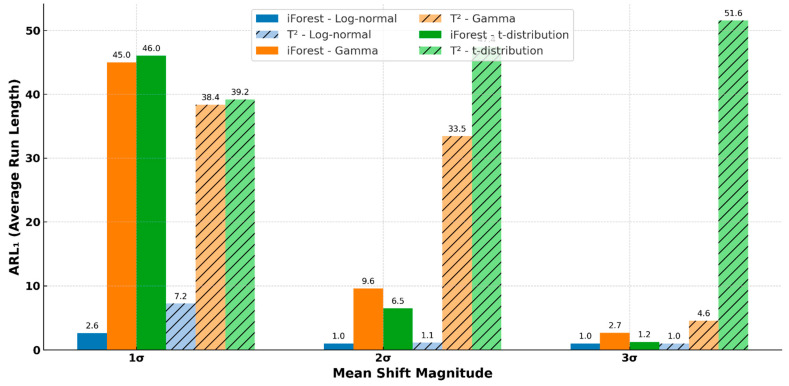
Comparison between iForest and Hotelling’s T^2^ on ${ARL}_{1}$.

iForest consistently outperformed T^2^ in terms of ${ARL}_{1}$ across all distributions and mean shifts (*t*(85) = −2.5, *p* = 0.015), as shown in Figure 5. Its strongest performance was observed in environments involving gamma and *t*-distributions and under small-to-medium mean shifts. This suggests that iForest’s advantage over T^2^ is particularly notable in the presence of skewed (log-normal) or heavy-tailed (*t*-distribution) data.

**Figure 6:**
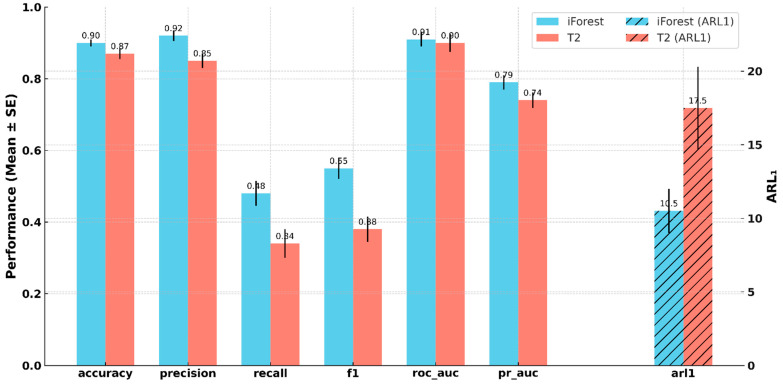
Performance comparison between iForest and Hotelling’s T^2^ across classification metrics. Error bars represent standard error. ${ARL}_{1}$ is plotted on a secondary axis.

iForest also outperformed T^2^ in all classification performance metrics, as illustrated in Figure 6. Among these metrics, accuracy, precision, recall, and F1-score were significantly higher for iForest (accuracy: *t*(129) = 2.46, *p* = 0.015; precision: *t*(105) = 2.47, *p* = 0.015; recall: *t*(129) = 2.46, *p* = 0.015; F1-score: *t*(125) = 2.76, *p* = 0.007), indicating a more consistent and reliable classification of normal versus abnormal cases. ROC AUC and PR AUC were also higher for iForest, though not statistically significant (ROC AUC: *t*(128) = 0.7, *p* = 0.49; PR AUC: *t*(134) = 1.32, *p* = 0.19). Finally, iForest exhibited a significantly lower ${ARL}_{1}$ than T^2^ (*p* < 0.05), reflecting faster average detection times.

## 5. Discussion and Conclusions

The results across all scenarios consistently demonstrate that the iForest-based control chart performs better in environments involving non-normal distributions (ARL_1_ for log-normal distribution: iForest = 1.5, T^2^ = 3.1) and small-to-medium mean shifts (ARL_1_ at 1 sigma mean shift: iForest = 2.6, T^2^ = 7.2). Its advantage is particularly pronounced in the presence of skewness (log-normal) or heavy tails (*t*-distribution). The robustness of iForest, coupled with its non-parametric and ensemble-based nature, allows it to adapt to a wider range of process behaviors than the covariance-based T^2^ chart. Moreover, while bootstrap resampling introduces minor performance variations, it does not substantially affect the comparative outcome, reinforcing iForest’s reliability as a distribution-free tool for modern process monitoring.

Evaluation results further showed that iForest outperformed T^2^ across all performance metrics, as summarized in Table 1, suggesting a more reliable and consistent classification. Precision, in particular, exhibited a strong margin, reflecting iForest’s lower FPR. These metrics displayed the most notable differences between the two algorithms. iForest also achieved significantly higher recall, underscoring its strength in detecting true anomalies. As a result, the F1-score—defined as the harmonic mean of precision and recall—was also significantly higher. Lastly, iForest exhibited a substantially lower ${ARL}_{1}$ than T^2^, indicating faster detection times, which is especially valuable in real-time or high-risk monitoring contexts.

**Figure 7:**
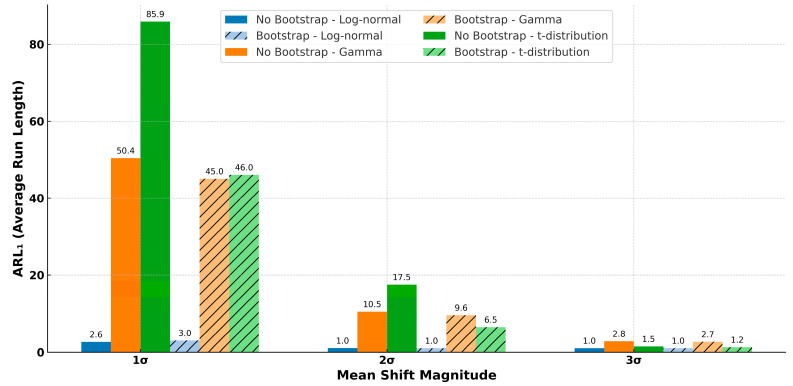
Effect of enabling bootstrap to iForest control chart on ${ARL}_{1}$.

This study found that enabling the bootstrap setting can lead to slightly improved performance across all classification metrics, as summarized in Figure 7. This implies that practitioners implementing iForest-based control charts should consider enabling bootstrap resampling when (1) the data likely follow heavy-tailed distributions or (2) detecting small process shifts is critical. Conversely, bootstrap resampling may be omitted in low-noise environments or when computational resources are limited without significantly compromising performance under larger process shifts.

The superiority of iForest was most evident in non-Gaussian contexts, such as log-normal and *t*-distributions, where the T^2^ chart exhibited significant detection delays in terms of ${ARL}_{1}$. Notably, under a 1σ mean shift in a log-normal distribution, iForest achieved an ${ARL}_{1}$ as low as 2.612, whereas the T^2^ chart yielded 7.232—highlighting a detection delay nearly three times longer. This result is expected, as the T^2^ chart is theoretically grounded in the assumption of multivariate normality [37]. These findings strongly suggest that iForest is a more suitable choice for processes involving non-Gaussian data distributions.

The result of this study showed that iForest outperformed the T^2^ chart in many scenarios. However, iForest has a limitation in detecting subtle process shifts, such as a 1σ mean shift. The reason for this limitation is that iForest calculates anomaly scores based on tree depth, which may not sufficiently capture the proximity of observations with tree cluster. To address this limitation, a distance or likelihood-based [38] scoring approach that combines tree depth with the Euclidean distance or likelihood to the centroid of each partitioned cluster could enhance the novelty detection performance. Integrating spatial closeness or likelihood within a cluster into the scoring mechanism may improve sensitivity to small process variations. However, to empirically investigate the effects of incorporating spatial proximity or likelihood, future studies are required.

Although we aimed to compare iForest with Hotelling’s T^2^, our scope remains limited. In multivariate monitoring, various techniques, such as exponentially weighted moving average (EWMA), cumulative sum control chart (CUSUM), local outlier factor (LOF), one-class support vector machine (one-class SVM), and principal component analysis (PCA)-based control charts, are widely used. As this study focused on examining the effects of varying data distributions and bootstrap setting in iForest, Hotelling’s T^2^ was selected as a benchmark method for comparison. For a more comprehensive evaluation, future studies can include these additional methods.

The bootstrap setting (true vs. false) may indirectly influence training through interaction with parameters, such as sample size and tree depth. Although enabling bootstrap does not alter the nominal sample size per tree, it introduces duplicate observations that can influence the tree structure. For example, bootstrapping may result in shallower trees due to early isolation of redundant samples. However, as iForest normalizes the anomaly score based on the expected path length, this potential side effect can be effectively compensated for. To draw a more definitive conclusion, a more comprehensive investigation should be conducted in future studies.

We selected critical simulation parameters (sample size, dimensionality, and degrees of freedom for the t-distribution) based on values widely adopted in previous anomaly detection and process monitoring studies. However, these parameters can substantially influence the behavior of iForest and the generalizability of our findings. For example, changes in dimensionality or the tail-heaviness of the distribution may affect both the separability of anomalies and the stability of anomaly scores. Although our parameter settings were grounded in ranges frequently reported in the literature [33,34,39,40,41], a more systematic investigation on the effects of these parameters is needed for better generalization of our findings.

Our experimental design focused on synthetic datasets generated from well-defined distributions. Although this approach inevitably limits the generalizability of our findings to real-world manufacturing data, it allowed for us to rigorously examine the intrinsic behavior of iForest under controlled conditions. This controlled investigation serves as a foundational benchmarking to better understand and improve iForest algorithm. Nonetheless, future studies should empirically validate our findings using real manufacturing data, which may further reveal strengths and limitations of iForest algorithm in practical applications.

## Figures and Tables

**Table 1:** Summary of evaluation results for iForest and T^2^.

Metric	iForest	T^2^	Statistical Significance
Accuracy	0.8942	0.8642	Yes
Precision	0.9088	0.8464	Yes
Recall	0.4790	0.3288	Yes
F1-score	0.5504	0.3834	Yes
ROC AUC	0.9034	0.8908	No
PR AUC	0.7880	0.7349	No
ARL_1_	10.3769	17.9133	Yes

## Data Availability

The data presented in this study are available on request from the corresponding author.

## Abbreviations

The following abbreviations are used in this manuscript:

ARL	Average run length
EWMA	Exponentially weighted moving average
EIF	Extended isolation forest
FPR	False positive rate
KNN	K-nearest neighbors
LOF	Local outlier factor
PCA	Principal component analysis
SPC	Statistical process control
SVDD	Support vector data description
